# Anisakiasis and Gastroallergic Reactions Associated with *Anisakis pegreffii* Infection, Italy

**DOI:** 10.3201/eid1903.121017

**Published:** 2013-03

**Authors:** Simonetta Mattiucci, Paolo Fazii, Alba De Rosa, Michela Paoletti, Angelo Salomone Megna, Antonio Glielmo, Maurizio De Angelis, Antonella Costa, Costantino Meucci, Vito Calvaruso, Italo Sorrentini, Giuseppe Palma, Fabrizio Bruschi, Giuseppe Nascetti

**Affiliations:** Author affiliations: Sapienza University of Rome, Rome, Italy (S. Mattiucci, A. De Rosa);; Umberto I Hospital, Rome (S. Mattiucci, M. De Angelis); Hospital S. Spirito, Pescara, Italy (P. Fazii);; Tuscia University, Viterbo, Italy (M. Paoletti, G. Nascetti);; G. Rummo Hospital, Benevento, Italy (A. Salomone Megna, A. Glielmo, I. Sorrentini);; Istituto Zooprofilattico Sperimentale della Sicilia, Palermo, Italy (A. Costa);; A. Maresca Hospital, Naples, Italy (C. Meucci);; F. Lotti Hospital, Pontedera, Pisa, Italy (V. Calvaruso);; The National Federation of Fishery Companies, Rome (G. Palma); University of Pisa, Pisa (F. Bruschi)

**Keywords:** *Anisakis pegreffii*, parasite, zoonosis, zoonoses, anisakiasis, allergic reaction, molecular diagnostic techniques, sequences analysis DNA, helminth antigens, gastroallergic anisakiasis

## Abstract

Human cases of gastric anisakiasis caused by the zoonotic parasite *Anisakis pegreffii* are increasing in Italy. The disease is caused by ingestion of larval nematodes in lightly cooked or raw seafood. Because symptoms are vague and serodiagnosis is difficult, the disease is often misdiagnosed and cases are understimated.

Human anisakiasis is a seafoodborne parasitic zoonosis caused by larval nematodes of the genus *Anisakis*. Humans are accidental hosts of the nematodes; they become infected by consuming raw or undercooked seafoods that harbor the nematode larvae in their flesh and muscle (*1*). The larvae do not further develop in humans; however, they can penetrate the gastrointestinal tract and form eosinophilic granulomas, often with pathologic consequences. There is a growing awareness that these parasites generate potentially life-threatening allergic reactions (*2*) when the live parasite attempts to penetrate the gastric mucosa. These reactions, termed gastroallergic anisakiasis, are characterized by urticaria, occurring generally on the arms and abdomen, and by angioedema or anaphylaxis (*3*).

The development of molecular tools for the diagnosis of human anisakiasis and greater awareness of this parasitic disease have led to an increase in the recorded number of cases of the disease during the past 20 years in many parts of the world (*4*). Two species of the genus *Anisakis* have been found to cause infections in humans: *A. simplex* sensu stricto and *A. pegreffii* (*1*), as has been confirmed by molecular markers (*4**–**7*). Although *Anisakis* spp. larvae are found in fish and squid worldwide, the prevalence of human infection is highest in countries where eating raw fish is widespread. However, the molecular identification of human cases is still scarce, especially in some European countries where allergic symptoms and hypersensitivity associated with the parasite have been reported (*2*). 

In this study, we report several new cases of gastric anisakiasis in Italy. Identifying the etiologic agent is challenging because *Anisakis* spp. larvae lack morphologic features that could be used to identify them at the species level. When larvae infest humans, they can become spoiled or fragmented, making it impossible to identify them at the genus level. We performed sequencing of nuclear and mitochondrial genes to identify the parasites and to gather data on the possible association between pathologic findings of human anisakiasis and different *Anisakis* spp. or haplotypes. In addition, serum samples from the patients were tested for IgE reactivity against specific antigens or allergens (IgE-As) of *A. pegreffii*.

## The Study

The 8 patients studied during June 2011–June 2012 were from the following regions of Italy: Abruzzo (*4*), Latium (*1*), Campania (*2*), and Tuscany (*1*). All of these patients experienced acute gastric pain and nausea for a period ranging from 2–3 hours to 2 days after they had eaten raw fresh marine fish (marinated anchovies). Three patients had allergic reactions that had different manifestations, but no anaphylactic shock occurred (Table). The parasites were observed penetrating the gastric wall in 2 patients.

**Table:** Clinical features of patients who had gastroallergic anisakiasis associated with *Anisakis pegreffii *infection, Italy*

Code	Home area of patient, province (region)	Location of nematode	Time since raw seafood ingested	Clinical features	Whole larval nematode or fragment, suspension, condition of specimen	DNA extraction method	Total IgE, IgE-As†
HuC1	Benevento (Campania)	Submucosal layer of gastric wall	2 d	Epigastric pain, edema of oral mucosa	Whole, 99% ethanol, very well conserved	CTAB	1,479, >100
HuC2	Pescara (Abruzzo)	Gastric mucosa	24 h	Epigastric pain, urticaria, generalized edema	Whole, 99% ethanol, well conserved	CTAB	2,180, >100
HuC3	Pescara (Abruzzo)	Lumen of stomach	24 h	Epigastric pain	Fragment, 99% ethanol, well conserved	CTAB	4,727, >100.0
HuC4	Pescara (Abruzzo)	Lumen of stomach	1 d	Epigastric pain, digestive symptoms	Fragment, 99% ethanol, well conserved	CTAB	511, 21.2
HuC5	Pescara (Abruzzo)	Lumen of stomach	1 d	Epigastric pain	Fragment 99% ethanol, well conserved	CTAB	Serum not available
HuC6	Rome (Latium)	Lumen of stomach	1 d	Epigastralgia, urticaria	Fragment 99% ethanol, well conserved	CTAB	Serum not available
HuC7	Pisa (Tuscany)	Lumen of stomach	2 d	Epigastric pain, vomiting	Fragment spoiled, 10% formalin, poorly conserved	Maxwell 16 Promega‡	2,062, 89.3
HuC8	Naples (Campania)	Lumen of stomach	2–3 h	Epigastralgia	Fragment spoiled, 10% formalin, poorly conserved	Maxwell 16 Promega	Serum not available

*Patients are indicated by their codes, HuC1–HuC8; all patients reported having eaten marinated anchovies before onset of illness. CTAB, cetyltrimethylammonium bromide (*8*). † Determined by ImmunoCAP ISAC diagnostic test (Phadia, Uppsala, Sweden). ‡Maxwell 16 Instrument System (Promega, Madison, WI, USA)

Nematodes and nematode fragments were endoscopically removed from the stomach and were stored in 99% ethanol or 10% formalin solutions. Total DNA was extracted from 2 mg of tissue from a single nematode or from a fragment removed from each patient (Table). We sequenced the mitochondrial gene (cytochrome c oxidase subunit II [*cox2*]) and nuclear genes (internal transcribed spacer [ITS] region, including ITS1, 5.8S, and ITS2 of rDNA). Amplification of the mitochondrial DNA (mtDNA) *cox2* gene (629 bp) and the ITS region (908 bp) of rDNA was performed on the 8 specimens as described (*8*,*9*). In addition, larval *Anisakis* specimens that were collected from anchovies (*Engraulis encrasicolus*) from the Tyrrhenian Sea, used for antigen preparation, were identified by using sequence analysis of the same genes.

Serum samples from patients HuC1, HuC2, HuC3, HuC4, and HuC7 were tested for IgE antibodies to *Anisakis* (IgE-As) by using ImmunoCAP (Phadia, Uppsala, Sweden). The IgE threshold level was defined by an antibody level of >0.35 kilounits of antibody per liter, as stated by the test manufacturer. Serum specimens were also analyzed by Western blot (WB) testing to detect specific IgE-As against antigens or allergens of *A. pegreffii*. For WB analysis, excretory or secretory antigens were obtained from larvae of *A. pegreffii* in stage 3 of 4 larval stages; the larvae were obtained from anchovies and cultured in vitro. 

Sequences of the ITS region of the rDNA (908 bp) obtained were aligned with those of *Anisakis* spp. stored in GenBank (http://www.ncbi.nlm.nih.gov/genbank/) by using ClustalX (www.clustal.org) as described (*10*). Overall, the highest nucleotide homology was observed with nucleotide homologs of *A. pegreffii*. Sequences from larval specimens showed 100% identity (GenBank accession nos. EU624343 and EU718479) with sequences available for *A. pegreffii*. Genetic variation was not observed in the 8 analyzed specimens. One sequence of the ITS region of rDNA, obtained during the current study, was deposited in GenBank under accession no. JQ900763. Similarly, the mtDNA *cox2* sequences obtained from the 8 nematodes or nematode fragments removed from the patients showed 99% homology to those deposited for *A. pegreffii*. 

**Figure 1:**
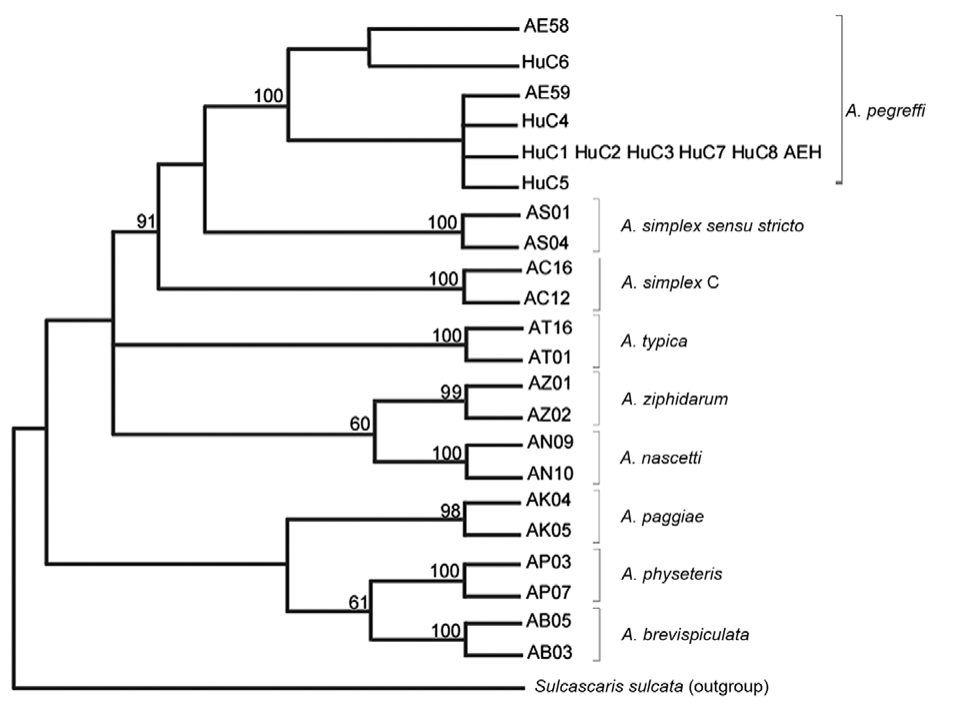
Maximum-parsimony bootstrap consensus tree inferred by PAUP* (*12*) for specimens of *Anisakis pegreffii* nematodes from patients with gastric anisakiasis (HuC1–HuC8) in Italy. Phylogenetic tree was obtained by mitochondrial DNA *cox2* sequences analysis (629 bp) of 1,000 pseudoreplicates related to *A. pegreffii* previously sequenced and deposited in GenBank. AEH indicates *A. pegreffii* associated with a previously reported case of intestinal anisakiasis *(7)*. Bootstrap values >70 are reported at the nodes. Sequences at the mitochondrial DNA *cox2* shown here have been deposited in GenBank under accession nos: JQ900759, JQ900760, JQ900761, and JQ900762.

A minor genetic differentiation (p-distance = 0.001) was found between *A. pegreffii* nematodes from the specimens isolated from humans and *A. pegreffii* nematodes from the Mediterranean Sea (*8*,*11*). In *A. pegreffii* (*7*), mtDNA cox2 sequences from 5 specimens (from patients HuC1, HuC2, HuC3, HuC7, and HuC8) corresponded to the most frequent haplotype, designated as H1. Sequences for patients HuC4, HuC5, and HC6 corresponded to 3 common haplotypes detected in *A. pegreffii* throughout its distribution range. Phylogenetic analysis performed by maximum-parsimony (*12*) showed that the larval specimens of *Anisakis* spp. nematodes from these 8 patients clustered in a well-supported clade, which includes specimens of *A. pegreffii* nematodes previously sequenced for the same gene (*7*,*8**,**11*) (Figure 1).

**Figure 2:**
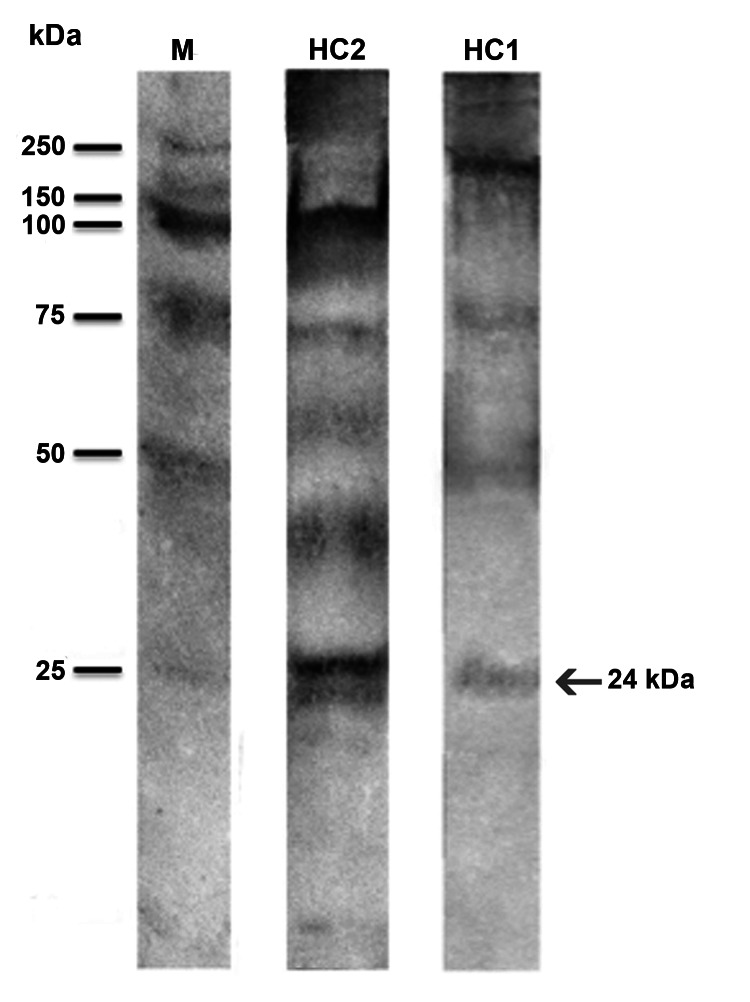
Western blot reaction of serum samples from patients HuC1 and HuC2 from Italy showing allergic reaction against *Anisakis pegreffii* antigens and allergens. M indicates molecular marker; arrow indicates the reaction at 24 kDa (Ani s1). IgE determination was performed with alkaline phosphatase conjugates obtained from goat anti-human IgE. Antigen-antibody binding was visualized by the alkaline phosphatase 5-bromo-4-chloro-3-indolyl phosphate p-nitroblue tetrazolium chloride system until bands appeared. Human serum specimens negative for *A. pegreffii* were used as controls. HC1 and HC2 represent patient identification numbers HuC1 and HuC2.

Serum specimens were available for 5 patients. In those specimens, a high level of IgE-As was found by using ImmunoCAP testing (Table). WB analysis revealed that 2 specimens (from patients HuC1 and HuC2) had IgE specific for the allergen Ani s1 at 24 kDa (Figure 2); the remaining serum specimens tested did not show reactivity.

## Conclusions

The first known case of anisakiasis among humans in Italy was described in 1996 (*13*). Since then, several cases of gastrointestinal anisakiasis have been reported there (*7*). The evident increase in the number of cases during recent years suggests that anisakiasis is an emergent zoonosis in Italy. In the present study, based on results of multiple gene sequence analyses, we identified several new cases of gastric anisakiasis caused by *A. pegreffii* nematodes. Among them, the mtDNA *cox2* sequence revealed a polymorphic gene, as previously shown in this group of parasites (*8*,*11*,*14*). Previously, the most common haplotype associated with gastric and intestinal anisakiasis in humans in Italy was designated as H1 (*7*). According to network analysis of the intraspecific genetic variation of the mtDNA *cox2* gene performed on *A. pegreffii* nematode populations from different geographic areas, H1 is likely the ancestral haplotype. This molecular marker could facilitate investigation of the possible association between mtDNA *cox2* haplotypes and pathologic features of anisakiasis.

Previous reports of allergic reaction related to *Anisakis* infections have not been associated with larval detection and identification of the parasite (*14*). Two serum specimens recognized Ani s1, a major secretory/escretory antigen/allergen of *Anisakis* spp., have been identified as the causal agent for 85% of allergic reactions (*2*,*3*). Gastroallergic anisakiasis associated with *A. pegreffii* nematodes was likely facilitated by a hypersensitivity reaction in those patients; the mechanism involved is probably an allergic reaction induced in the submucosal layer of the gastric wall around the penetration site of the helminth. The high level of IgE-As observed in the remaining serum specimens was likely related to cross-reactive antibodies against *Anisakis* antigens considered to be panallergens (*3*).

The public should be cautioned against eating raw marinated anchovies, the main source of human anisakiasis in Italy, and other raw or lightly cooked seafoods. Clinicians should be made aware of the potential risk for severe allergic reactions in patients with gastric anisakiasis, and encouraged to make and report molecular identification of the helminths to increase knowledge about the association between different *Anisakis* spp. and their pathogenic effects on humans. 
